# Congenital Cystic Adenomatoid Malformation (CCAM) Type II: A Rare Case of Sudden Infant Death

**DOI:** 10.3390/children9121830

**Published:** 2022-11-26

**Authors:** Monica Salerno, Francesco Sessa, Giuseppe Cocimano, Salvatore Roccuzzo, Massimiliano Esposito, Cristoforo Pomara

**Affiliations:** Department of Medical, Surgical and Advanced Technologies, “G.F. Ingrassia” University of Catania, 95121 Catania, Italy

**Keywords:** congenital cystic adenomatoid malformation (CCAM), congenital pulmonary airway malformation (CPAM), sudden infant death, multidisciplinary approach

## Abstract

Congenital cystic adenomatoid malformation (CCAM) is a developmental lesion of the lungs and terminal respiratory structures, which is characterized by pseudocysts, lesions, and cystically dilated airways. CCAM is also known as congenital pulmonary airway malformation (CPAM). Various classification systems for CCAM have been described, and based on a recent classification, CCAMs are classified morphologically into five different types (Type 0, I, II, III, and IV) based on lesion sizes. The most common manifestation of CCAM in neonates and children is respiratory distress (RD). Spontaneous pneumothorax is a rare manifestation of CCAM. In this case report, we discuss a CCAM type-II case of a 38-day-old female infant with a radiological post-mortem diagnosis of a large left-side spontaneous pneumothorax. The gross examination of the lungs revealed multiple emphysematous air bubbles up to 0.5 cm in diameter, and the histological examination revealed focal pleural fibrosis, accompanied by thickened septa and atelectasis. In this scenario, the routine use of prenatal ultrasonography would be important to obtain a timely prenatal diagnosis. At the same time, improvements in surgical techniques, as well as greatly improved imaging techniques, have improved the outcome of these patients. Finally, it is important to remark on the importance of autopsy in the case of sudden infant death with a suspected CCAM.

## 1. Introduction

Congenital cystic adenomatoid malformation (CCAM) is an unusual form of cystic disease of the lungs, also known as congenital pulmonary adenomatoid malformation (CPAM). It is due to a developmental anomaly of the terminal respiratory structures [1]. The incidence of CCAM is estimated at 1:25,000–35,000 pregnancies [2]. CCAM commonly manifests as respiratory distress (RD) in neonates and infants [1]. CCAM has been described in association with bronchopulmonary sequestration, extralobar intra-abdominal sequestration, and bronchial atresia in both live births and stillbirths. Furthermore, a close association between CCAM and polyhydramnios has been reported in a few cases. Several classification systems have been developed to describe the morphology of these malformations. CCAMs can be classified using the Stocker classification system, which distinguishes lesions based on diameter and predominant cell types [3,4].

When following the Stocker classification system, CCAM may be subdivided into three types, based on gross and microscopic criteria: type I is usually associated with a good prognosis and includes multiple large cysts (>2 cm in diameter) or a single large cyst, surrounded by numerous smaller cysts. Type II represents over 40% of all CCAM cases, and is associated with multiple small cysts (<1 cm in diameter), lined with ciliated cuboidal to columnar epithelium; in addition, striated muscles can occasionally be found. This form is usually associated with other congenital abnormalities, affecting the prognosis. CCAM Type III is rare; cysts are too small to see at gross examination (<0.5 cm in diameter) and the lung has a sponge-like appearance. The cysts are lined with non-ciliated cuboidal or columnar epithelium; this type represents about 5% of all cases [5]. However, based on a more recent classification, CCAMs are now classified morphologically into five different types: in type 0, the development is arrested at the stage of tracheal/bronchial formation (tracheal epithelium, also in the presence of cartilage), and the cysts are usually < 0.5 cm. In type I, the development is arrested at the stage of bronchia formation. In the bronchial epithelium, cartilage is rarely present, with a squamous-like epithelium; the cyst dimension is ranked from 4 to 10 cm. In type II, the development of the bronchial tree is arrested at the glandular stage; the presence of multiple cysts < 2.5 cm, covered with columnar epithelium, is usually described. In type III, the development of the bronchial tree is arrested at the glandular stage, generating multiple cysts of < 1.5 cm, covered with columnar epithelium, which represents a typical adenomatoid malformation. In type IV, the development of the bronchial tree is arrested at the stage of bronchial formation (acinar epithelium); cysts are usually about 2–4 cm (pleuropulmonary blastoma—PPB) and cartilage is absent [2,6,7,8].

Microscopically, the lesions may not be classified as true cysts, considering the fact that they communicate with the surrounding parenchyma. In rare cases, there is evidence of malignant transformation, usually into bronchioloalveolar carcinoma (BAC); sarcomatous and blastomatous malignant transformation have also been reported, although they are considered rare in adults [1,2,9]. The hamartomatous nature of abnormal lung tissue may include solid areas as well as cystic areas, characterized by the proliferation of bronchiolar-like structures, interconnected with cystic structures of various sizes that lack normal alveoli [10]. This is observed in 80–85% of full-term and pre-term neonates. These lesions are seldom observed in children between the ages of 2 and 10 years, as well as in adolescents and adults. CCAM usually presents unilaterally and is restricted to the lower lobe, with rare occurrences on the lower left lobe. A common presentation in neonates is RDS. However, spontaneous pneumothorax is a rare manifestation of CCAM [11,12,13].

Lung lesions are mainly diagnosed nowadays by prenatal ultrasound imaging, but magnetic resonance imaging can be very useful in the differential diagnosis of bronchogenic cysts, bronchial atresia, or multilobe involvement [10].

Considering that this is a rare pathology, in this article, we report the case of an infant who was otherwise healthy until she presented with a fatal spontaneous pneumothorax as the initial manifestation of CCAM at 5 weeks of age. Through this case presentation, a further goal is to discuss CCAM, highlighting the current strengths and weaknesses of the forensic approach.

## 2. Case Presentation

**Figure 1:**
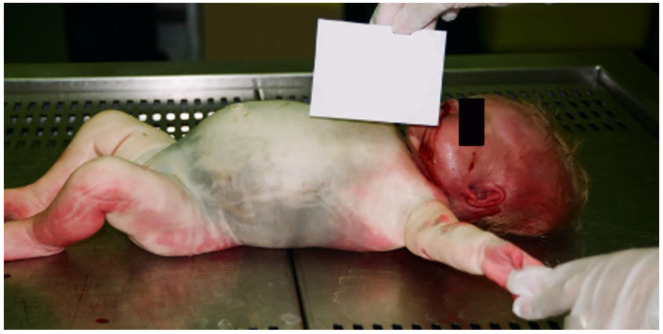
External examination revealed a putrefaction-induced green discoloration of the skin of the trunk and abdominal bloating due to decomposition gas.

A healthy 38-day-old female infant, born at full-term, was found dead in her crib by her parents. At birth, the newborn was small for her gestational age (SGA), weighing 2530 g, with a length of 46 cm and a head circumference of 32 cm. She was discharged 7 days after birth, in good health. After the discovery of her death, the parents alerted the emergency medical services. The local prosecutor’s officer ordered a full autopsy, to determine the cause of death and the time since death. The autopsy was performed 7 days later. External examination revealed a putrefaction-induced green discoloration of the skin of the thorax, abdomen, and perineum (Figure 1). No ventilation or intubation was carried out on the baby girl. The subcutaneous emphysema was due to putrefactive gases; the autopsy was performed several days after death, and it was midsummer. The body weight was 2970 g, the crown-to-heel length was 49 cm, and the foot length was 7 cm (Figure 1).

**Figure 2:**
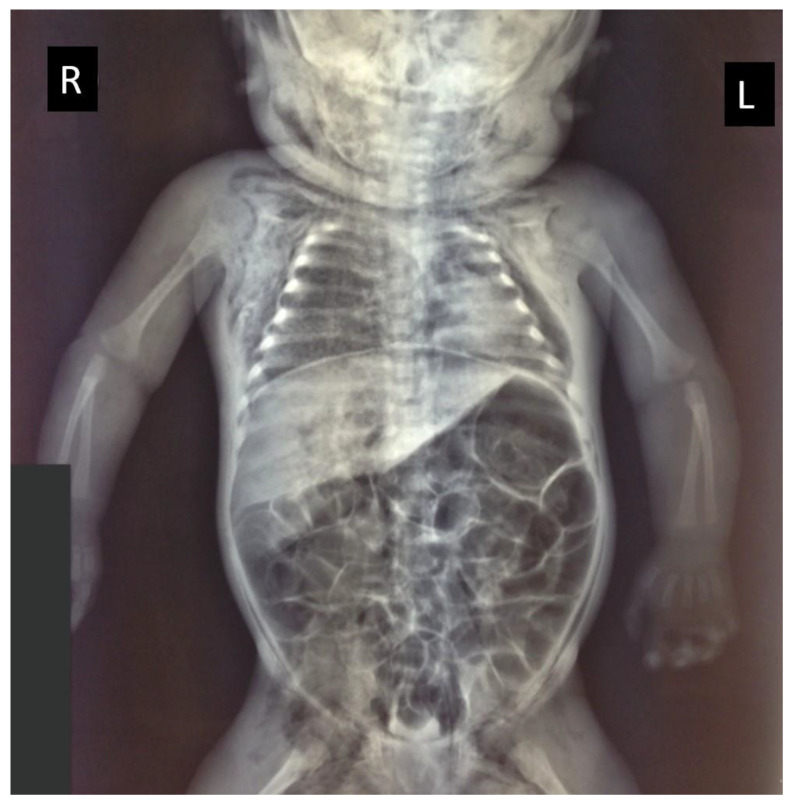
Radiological investigations demonstrated skin emphysema on the right and left side of the neck, the thorax, and in the groin area, with a large left-sided pneumothorax and compression of the collapsed lung (R = right side; L = left side) and a bloating intestine.

The chest X-ray confirmed the absence of skeletal injuries and revealed a large pneumothorax, with a collapsed left lung and a mediastinal shift (Figure 2).

**Figure 3:**
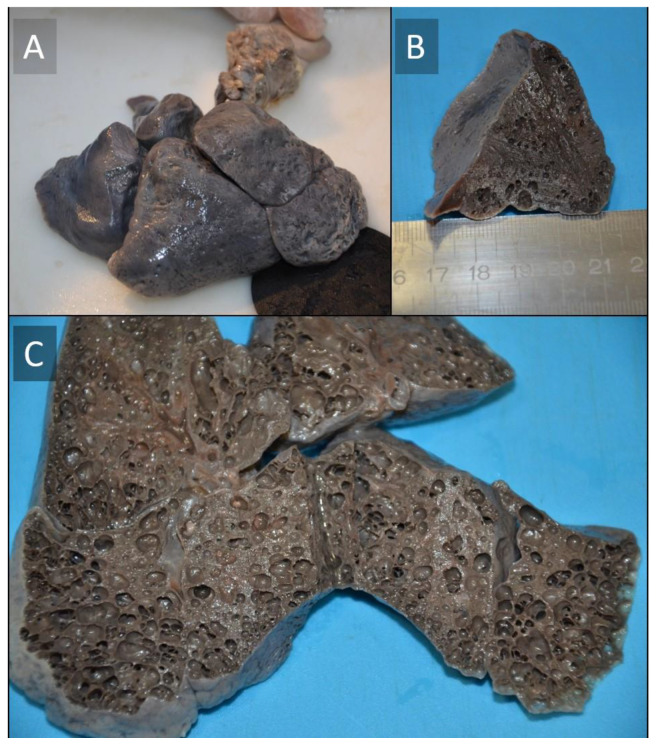
Macroscopic aspect of the lungs (**A**–**C**). Both lungs appeared to be diffusely enlarged and the consistency was firmer than usual. Externally, a brownish coloration was diffusely distributed on all lobes. The cut surface was solid. The combined weight of the lungs (39 g) was slightly below normal.

The autopsy was performed according to the Letulle method [14]. No biological fluids were found in the pleural or abdominal cavities. The autopsy excluded the occurrence of acute and significant macroscopic abnormalities in all organs except the lungs. After formalin fixation, at the gross examination, both lungs appeared to be diffusely enlarged, with a firm consistency. A careful examination revealed copious emphysematous air bubbles on the pleural surfaces, with diffuse crackling upon palpation. The gross section of the lungs revealed a diffuse brownish substance over the porous lung parenchyma, with multiple bubbles. The bubbles were attributed to a large collection of numerous cysts measuring up to 0.5 cm in diameter (Figure 3).

### Histopathological Investigation

**Figure 4:**
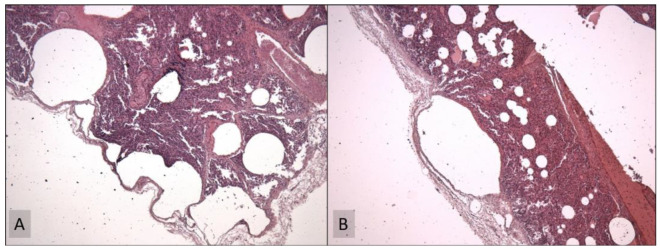
Histological examination (hematoxylin and eosin (H&E) staining, at 40×) of cystic lesions at low magnification: the cysts were lined by columnar epithelium in the absence of smooth muscle or striated muscle in the walls of the cysts (A). Focal pleural fibrosis and atelectasis of the parenchyma were exhibited, alternating with areas of acute and chronic emphysema, characterized by alveolar cavities (B).

The etiopathogenesis was defined by histological examinations that were performed on lung tissue samples, using hematoxylin and eosin (H&E) staining. Histological examinations performed on lung parenchyma samples revealed the presence of focal pleural fibrosis, accompanied by thickened septa and atelectasis. Moreover, areas of acute and chronic emphysema, characterized by cavitation with pleural air bubbles and subpleural alveolar dilatations, were also present (Figure 4).

There was no evidence of inflammatory involvement. The histological examination of the other organs was unremarkable. Post-mortem examination attributed the cause of death to acute respiratory failure, initiated by a type-II CCAM-induced spontaneous pneumothorax.

## 3. Discussion

CCAM cases can be classified into several types. Type I (the macrocystic or large cyst type) accounts for over 50% of all cases, consisting of single or multiple cysts, which are usually large in size (2 to 10 cm in diameter) and few in number (one to four). The cysts can cause a mediastinal shift or even hypoplasia of the adjacent lung and can affect the bronchial tree [15,16,17]. The cysts are lined with pseudostratified columnar epithelium, the differentiation of which may sometimes include goblet cells [18]. These cases generally have a good prognosis; however, cysts comprising one-third of the lung volume can grow rapidly and unpredictably. Based on data from the literature [19,20,21], the main characteristics of interstitial lung disease in infants and young children (chILD) of < 2 years are summarized in Table 1.

As confirmed in our case study, chest radiography can be considered fundamental in the diagnosis of suspected congenital cystic lung disease in childhood [2,9,11,22]. Chest radiography is usually enough to identify CCAM cases, especially when the cysts are large enough to cause clinical problems. As demonstrated in the discussed case, the usual appearance is a mass containing air-filled cysts. Other radiologic findings that may lead clinicians to a CCAM diagnosis are mediastinal displacement, pleural and pericardial effusions, and pneumothorax. In particular cases, the diagnosis may not be performed exclusively with chest radiographs, especially when a mass is detected, without highlighting the presence of cysts. In these cases, chest CT provides safe and rapid support in CCAM diagnosis [23,24,25,26]. CT usually assumes an important role when multilocular thin-walled (from imperceptible, up to < 4 mm) cystic lesions surrounded by normal-appearing lung parenchyma are found. In addition, the possible presence of an infection in the same area of the lesion, as well as the presence of effusions, may complicate CCAM diagnosis. Moreover, a CT scan may be considered an important diagnostic tool because it can highlight additional coexisting lesions. Another important technique that can be used for diagnostic purposes, especially to distinguish between microcystic and macrocystic lesions, is high-resolution chest tomography (HRCT) [27,28,29,30].

Autopsy remains the gold standard method to establish the cause of death: knowledge of the cause of death may be critically important, both in health policies and in penal diatribes [31,32]. In the case of a post-mortem investigation, the histologic findings may be considered mandatory to define the exact cause of death, particularly in the case of sudden infant death [33,34,35]. CCAM has been defined as a hamartoma, with an excess of one or more tissue components. Cysts may communicate with the bronchial tree and derive their blood supply from the pulmonary circulation [36,37].

Based on the literature data, macrocystic lesions (cysts > 5 mm) are usually associated with a good prognosis, considering the fact that they do not generate hydrops; on the contrary, microcystic lesions (cysts < 5 mm) are usually associated with a poor prognosis, generating fetal hydrops [38,39]. Another important consideration concerns other congenital defects that could be associated with CCAM, for instance, bilateral renal agenesis, hydrocephalus, gastrointestinal or abdominal wall defects (diaphragmatic hernia, jejunal atresia, and tracheoesophageal fistula), spinal deformities (cervical spine/thoracic spine), epiaortic vessels and myocardial anomalies (truncus arteriosus and tetralogy of Fallot), and sirenomelia [40]. Patients with CCAM may present with clinical features of pneumothorax in the early neonatal period [41,42]. Huang et al. reported that different factors are independently associated with a poor outcome; for example, gestational age at birth, birthweight, Apgar score, and the diagnosis time could be considered fundamental to the survival rate [38]. As recently discussed by Barikbin et al., postnatal lung function tests are a useful tool to detect and monitor CCAM, allowing a timely decision for or against surgical intervention [43].

Nevertheless, despite the importance of this theme, to date, no guidelines are available concerning the management of these patients, especially regarding prophylactic surgery in asymptomatic patients or in terms of proceeding with a conservative follow-up. In this way, it is important to highlight the European experience: in 2016, the congenital lung anomalies (CLA) Swiss database (CLADatabase) was created, with the aim of recording and collecting surgical lung samples, thus improving our knowledge regarding patient management [44].

## 4. Conclusions

In this scenario, the routine use of prenatal ultrasonography is important to obtain a timely prenatal diagnosis: this could be fundamental in cases of familiarity with this pathology. Moreover, advances in prenatal and postnatal surgical techniques, as well as greatly increased imaging techniques, have improved the outcome of these patients and their quality of life. In particular, postnatal intervention is dictated by clinical status at birth. In asymptomatic cases, postnatal investigation consists of a plain radiographic evaluation on the first day after delivery, along with a chest CT scan within 1 month of birth [45]. Symptomatic lesions require urgent radiological evaluation with chest radiography, followed by surgical excision. The management of symptomatic lesions carries higher morbidity [46]. Although a series of studies indicate that the technique was performed safely on patients who were asymptomatic at birth [47], surgical excision is still controversial, with some centers opting for conservative management [48,49].

Finally, it is important to remark upon the leading role of autopsy in cases of sudden infant death with suspected CCAM: post-mortem investigation remains the most important method to ascertain the exact cause of death for the purposes of penal and civil litigation. Moreover, future studies should be performed to define guidelines for the diagnosis and management of CCAM.

## Figures and Tables

**Table 1:** Interstitial lung disease in infants and young children (chILD) of < 2 years.

Developmental diseases	Genetic mutations
Congenital cystic adenomatoid malformation (CCAM) Acinar dysplasia Alveolar capillary dysplasia (ACD)	SFTPB (surfactant protein B deficiency) SFTPC (surfactant protein C deficiency) CSF2RA (pulmonary alveolar proteinosis/PAP) CSF2RB (pulmonary alveolar proteinosis/PAP) NKX2-1 (interstitial lung disease) ABCA-3 (surfactant deficit)
**Growth disorders**	**Undefined etiology**
Pulmonary hypoplasia Congenital heart disease Filamin A deficiency Chronic neonatal lung disease (prematurity leading to bronchopulmonary dysplasia) Acquired chronic lung disease in term infants	Autoimmune Storage diseases Langerhans cell histiocytosis Pulmonary interstitial glycogenosis (PIG) Neuroendocrine cell hyperplasia in infancy (NEHI)

## Data Availability

All data are included in the main text.

## References

[1] Sirithangkul S., Chuengchitraks S., Staworn D., Laohapand C., Silarat T. (2010). Late Manifestation of Congenital Cystic Adenomatoid Malformation with Lung Abscess: A Case Report. J. Med. Assoc. Thail..

[2] Strumillo B., Jóźwiak A., Palka A., Szaflik K., Piaseczna-Piotrowska A. (2018). Congenital Cystic Adenomatoid Malformation-Diagnostic and Therapeutic Procedure: 8-Year Experience of One Medical Centre. Kardiochirurgia i Torakochirurgia Polska.

[3] Viana R., Carvalho L., Santos C. (2022). Congenital Pulmonary Airway Malformation: A Rare Diagnosis in Adulthood. Respirol. Case Rep..

[4] Koga H., Ochi T., Hirayama S., Watanabe Y., Ueno H., Imashimizu K., Suzuki K., Kuwatsuru R., Nishimura K., Lane G.J. (2021). Congenital Pulmonary Airway Malformation in Children: Advantages of an Additional Trocar in the Lower Thorax for Pulmonary Lobectomy. Front. Pediatr..

[5] Stocker J.T., Madewell J.E., Drake R.M. (1977). Congenital Cystic Adenomatoid Malformation of the Lung. Classification and Morphologic Spectrum. Hum. Pathol..

[6] Widiyanto M.K., Ismail D., Hayuningrat P.K., Muhammad F. (2021). Congenital Cystic Adenomatoid Malformation Type i in a Newborn with Sepsis: A Case Report. Malays. J. Med. Health Sci..

[7] Kotecha S., Barbato A., Bush A., Claus F., Davenport M., Delacourt C., Deprest J., Eber E., Frenckner B., Greenough A. (2012). Antenatal and Postnatal Management of Congenital Cystic Adenomatoid Malformation. Paediatr. Respir. Rev..

[8] Stocker J.T. (2009). Cystic Lung Disease in Infants and Children. Fetal. Pediatr. Pathol..

[9] Argeitis J., Botsis D., Kairi-Vassilatou E., Hasiakos D., Papakonstantinou K., Kondi-Pafiti A. (2008). Congenital Cystic Adenomatoid Lung Malformation: Report of Two Cases and Literature Review. Clin. Exp. Obstet. Gynecol..

[10] Yamashita A., Hidaka N., Yamamoto R., Nakayama S., Sasahara J., Ishii K., Mitsuda N. (2015). In Utero Resolution of Microcystic Congenital Cystic Adenomatoid Malformation after Prenatal Betamethasone Therapy: A Report of Three Cases and a Literature Review. J. Clin. Ultrasound.

[11] Miller J.A., Corteville J.E., Langer J.C. (1996). Congenital Cystic Adenomatoid Malformation in the Fetus: Natural History and Predictors of Outcome. J. Pediatr. Surg..

[12] Kersten C.M., Hermelijn S.M., Mullassery D., Muthialu N., Cobanoglu N., Gartner S., Bagolan P., Mesas Burgos C., Sgrò A., Heyman S. (2022). The Management of Asymptomatic Congenital Pulmonary Airway Malformation: Results of a European Delphi Survey. Children.

[13] Mardy C., Blumenfeld Y.J., Arunamata A.A., Girsen A.I., Sylvester K.G., Halabi S., Rubesova E., Hintz S.R., Tacy T.A., Maskatia S.A. (2020). In Fetuses with Congenital Lung Masses, Decreased Ventricular and Atrioventricular Valve Dimensions Are Associated with Lesion Size and Clinical Outcome. Prenat. Diagn..

[14] Pomara C., Fineschi V., Pomara C., Fineschi V. (2020). Forensic and Clinical Forensic Autopsy. An Atlas and Handbook—2nd Edition.

[15] Mackenzie T.C., Guttenberg M.E., Nisenbaum H.L., Johnson M.P., Adzick N.S. (2001). A Fetal Lung Lesion Consisting of Bronchogenic Cyst, Bronchopulmonary Sequestration, and Congenital Cystic Adenomatoid Malformation: The Missing Link?. Fetal Diagn. Ther..

[16] Seear M., Townsend J., Hoepker A., Jamieson D., McFadden D., Daigneault P., Glomb W. (2017). A Review of Congenital Lung Malformations with a Simplified Classification System for Clinical and Research Use. Pediatr. Surg. Int..

[17] Müller A.M. (2015). Pathology of Pediatric Lung Diseases and Malformations|Pathologie von Pädiatrischen Lungenerkrankungen Und Fehlbildungen. Pneumologe.

[18] Annam V., Korishetty S.I., Yelikar B.R., Hippargi S.B., Shivalingappa D.B. (2010). Bilateral Congenital Cystic Adenomatoid Malformation, Stocker Type III with Associated Findings and Review of Literature. Indian J. Pathol. Microbiol..

[19] Kurland G., Deterding R.R., Hagood J.S., Young L.R., Brody A.S., Castile R.G., Dell S., Fan L.L., Hamvas A., Hilman B.C. (2013). An Official American Thoracic Society Clinical Practice Guideline: Classification, Evaluation, and Management of Childhood Interstitial Lung Disease in Infancy. Am. J. Respir. Crit. Care Med..

[20] Deutsch G.H., Young L.R., Deterding R.R., Fan L.L., Dell S.D., Bean J.A., Brody A.S., Nogee L.M., Trapnell B.C., Langston C. (2007). Diffuse Lung Disease in Young Children: Application of a Novel Classification Scheme. Am. J. Respir. Crit. Care Med..

[21] Nogee L.M. (2017). Interstitial Lung Disease in Newborns. Semin. Fetal Neonatal Med..

[22] Khan H., Kurup M., Saikia S., Desai A., Mathew M., Sheikh A., Goonasekera C.D.A. (2021). Morbidity after Thoracoscopic Resection of Congenital Pulmonary Airway Malformations (CPAM): Single Center Experience over a Decade. Pediatr. Surg. Int..

[23] Hermelijn S.M., Mackenbach M.J., van Horik C., Ciet P., Wolf J.L., von der Thüsen J.H., Wijnen R.M.H., Tiddens H.A.W.M., Schnater J.M. (2022). Quantitative CT Imaging Analysis to Predict Pathology Features in Patients with a Congenital Pulmonary Airway Malformation. J. Pediatr. Surg..

[24] Ibrahim H., Hafiz A., Rabiu B., Abdullahi U., Ghidazuka Y., Aliyu I. (2019). Spontaneous Resolution of a Congenital Multicystic Lung Lesion in a Newborn. Med. J. Dr. D.Y. Patil Vidyapeeth.

[25] Garg S., Singh R.S., Singh H. (2018). Congenital Cystic Adenomatoid Malformation of the Lung in Adults: Report of Two Cases and Review of the Literature. Indian J. Thorac. Cardiovasc. Surg..

[26] Sharma A., Pannu M.S., Lata N., Dhillon S.P.S., Singh N., Sareen A. (2016). Rare Presentation of Congenital Cystic Adenomatoid Malformation of the Lung. J. Nepal Paediatr. Soc..

[27] Badour M., Hussain B., Hammed A., Sawssan A., Saeed F. (2021). A Rare Case of Congenital Cystic Adenomatoid Malformation: Mimics Pneumonia Manifestations. Ann. Med. Surg..

[28] Hermelijn S.M., Elders B.B.L.J., Ciet P., Wijnen R.M.H., Tiddens H.A.W.M., Schnater J.M. (2021). A Clinical Guideline for Structured Assessment of CT-Imaging in Congenital Lung Abnormalities. Paediatr. Respir. Rev..

[29] Zhang Z.-J., Huang M.-X. (2015). Children with Congenital Cystic Adenomatoid Malformation of the Lung CT Diagnosis. Int. J. Clin. Exp. Med..

[30] Sessa F., Esposito M., Messina G., di Mizio G., di Nunno N., Salerno M. (2021). Sudden Death in Adults: A Practical Flow Chart for Pathologist Guidance. Healthcare.

[31] di Nunno N., Patanè F.G., Amico F., Asmundo A., Pomara C. (2020). The Role of a Good Quality Autopsy in Pediatric Malpractice Claim: A Case Report of an Unexpected Death in an Undiagnosed Thymoma. Front. Pediatr..

[32] Schiavone S., Mhillaj E., Neri M., Morgese M.G., Tucci P., Bove M., Valentino M., di Giovanni G., Pomara C., Turillazzi E. (2017). Early Loss of Blood-Brain Barrier Integrity Precedes NOX2 Elevation in the Prefrontal Cortex of an Animal Model of Psychosis. Mol. Neurobiol..

[33] Neri M., Othman S.M., Cantatore S., de Carlo D., Pomara C., Riezzo I., Turillazzi E., Fineschi V. (2012). Sudden Infant Death in an 8-Month-Old Baby with Dengue Virus Infection: Searching for Virus in Postmortem Tissues by Immunohistochemistry and Western Blotting. Pediatr. Infect. Dis. J..

[34] Bertozzi G., Maglietta F., Baldari B., Besi L., Torsello A., di Gioia C.R.T., Sessa F., Aromatario M., Cipolloni L. (2020). Mistrial or Misdiagnosis: The Importance of Autopsy and Histopathological Examination in Cases of Sudden Infant Bronchiolitis-Related Death. Front. Pediatr..

[35] Santacroce R., Santoro R., Sessa F., Iannaccaro P., Sarno M., Longo V., Gallone A., Vecchione G., Muleo G., Margaglione M. (2008). Screening of Mutations of Hemophilia A in 40 Italian Patients: A Novel G-to-A Mutation in Intron 10 of the F8 Gene as a Putative Cause of Mild Hemophilia a in Southern Italy. Blood Coagul. Fibrinolysis.

[36] Sintim-Damoa A., Cohen H.L. (2022). Fetal Imaging of Congenital Lung Lesions with Postnatal Correlation. Pediatr. Radiol..

[37] Zhu P., Cheng K., He M., Wang Y., Shen P., He K., Xu C., Zhang B., Liu Z. (2022). Diagnostic Value of Congenital Pulmonary Airway Malformation Volume Ratio for Fetal Hydrops Due to Congenital Lung Malformations: A Systematic Review and Meta-Analysis. Orphanet J. Rare Dis..

[38] Huang Y.-Y., Chang Y.-J., Chen L.-J., Lee C.-H., Chen H.-N., Chen J.-Y., Chen M., Hsiao C.-C. (2022). Survival of Hydrops Fetalis with and without Fetal Intervention. Children.

[39] Jeong B.-D., An S.-A., Lee M.-Y., Won H.-S., Han M., Yoon H., Lee J.-H., Cho Y.-J. (2020). Comparison of the Prognostic Factors of Fetuses with Congenital Pulmonary Airway Malformations According to Type. J. Ultrasound Med..

[40] Mon R.A., Johnson K.N., Ladino-Torres M., Heider A., Mychaliska G.B., Treadwell M.C., Kunisaki S.M. (2018). Diagnostic Accuracy of Imaging Studies in Congenital Lung Malformations. Arch. Dis. Child. Fetal Neonatal Ed..

[41] Chaouachi S., ben Hamida E., ben Fraj N., Blibèche S., Marrakchi Z. (2011). Congenital Cystic Adenomatoid Malformation of the Lung: Two Cases Report. Tunis. Med..

[42] Shupe M.P., Kwon H.P., Morris M.J. (2014). Spontaneous Pneumothorax in a Teenager with Prior Congenital Pulmonary Airway Malformation. Respir. Med. Case Rep..

[43] Barikbin P., Roehr C.C., Wilitzki S., Kalache K., Degenhardt P., Bührer C., Schmalisch G. (2015). Postnatal Lung Function in Congenital Cystic Adenomatoid Malformation of the Lung. Ann. Thorac. Surg..

[44] Isabellea V., Wildhaber Barbara E., Ueli M., Nicolas R., Daniel T., Dietmar C., Juerg B., Constance B.-A., Isabelle R.-M. (2019). A Swiss Database and Biobank to Better Understand and Manage Congenital Lung Anomalies. Swiss Med. Wkly..

[45] Davenport M., Warne S.A., Cacciaguerra S., Patel S., Greenough A., Nicolaides K. (2004). Current Outcome of Antenally Diagnosed Cystic Lung Disease. J. Pediatr. Surg..

[46] Stanton M., Davenport M. (2006). Management of Congenital Lung Lesions. Early Hum. Dev..

[47] Tsai A.Y., Liechty K.W., Hedrick H.L., Bebbington M., Wilson R.D., Johnson M.P., Howell L.J., Flake A.W., Adzick N.S. (2008). Outcomes after Postnatal Resection of Prenatally Diagnosed Asymptomatic Cystic Lung Lesions. J. Pediatr. Surg..

[48] Jaffé A., Chitty L.S. (2006). Congenital Cystic Adenomatoid Malformations May Not Require Surgical Intervention. Arch. Dis. Child. Fetal Neonatal Ed..

[49] Chetcuti P.A.J., Crabbe D.C.G. (2006). CAM Lungs: The Conservative Approach. Arch. Dis. Child Fetal Neonatal Ed..

